# Comparative estimates are essential, not one methodological option among many

**DOI:** 10.1093/esj/aakag093

**Published:** 2026-07-29

**Authors:** Victor Volovici, Mervyn D I Vergouwen

**Affiliations:** Department of Neurosurgery, Erasmus MC University Medical Centre, Rotterdam, The Netherlands; Center for Complex Microvascular Surgery, Erasmus MC University Medical Centre, Rotterdam, The Netherlands; Department of Neurology and Neurosurgery, UMC Utrecht Brain Centre, University Medical Centre Utrecht, Utrecht University, Utrecht, The Netherlands

**Keywords:** causal inference, confounding by indication, evidence standards, intracranial aneurysm, neurovascular devices, randomised controlled trials

Dear editor,

We thank Dr Taschner and colleagues for their interest in our editorial. We are pleased that they agree that many published studies on intracranial aneurysm treatment suffer from methodological limitations, incomplete outcome reporting and insufficient follow-up, and we share their support for greater transparency, independent outcome assessment, protocol registration and standardised outcomes. We respectfully disagree, however, with their critical view of randomised controlled trials.

The primary aim of intracranial aneurysm treatment is to maximise the number of life years with good quality of life, which requires balancing the risk of rupture against that of treatment complications. No device used to treat intracranial aneurysms is intrinsically safe or effective; every device, even in expert hands, carries a risk of complications. High-quality comparative evidence is therefore needed to quantify treatment-related risks and establish the relative safety and effectiveness of available devices, so that clinicians can select the treatment offering the best balance of benefits and harms for the individual patient.

To describe a device as safe is not simply to assert that adverse events are less frequent than, or similar to, those of an alternative. Safety depends on the absolute risk of treatment-related harm and on the balance of that risk against the expected benefit in reducing (re)rupture, and assessing this balance requires valid comparative estimates. A single-arm registry with independent adjudication, prespecified analysis plans and consecutive enrolment can rigorously describe outcomes in treated patients, yet it cannot supply the comparator needed to interpret them. The empirical record is ironclad: a substantial proportion of highly cited observational claims of clinical benefit are contradicted or attenuated when subsequently tested in randomised controlled trials.^1^

Interventional neuroradiology is no exception. Advanced endovascular devices have been described as safe and effective on the basis of single-arm studies and observational experience, often before comparative data existed. Such studies cannot establish whether a device confers greater net benefit than existing strategies, because they cannot account for what would have occurred under alternative management. Expert opinion, regulatory approval and early clinical adoption should therefore be complemented by robust comparative evidence before claims of safety and effectiveness are made.

Dr Taschner et al. propose a “proportionate” approach in which evidentiary requirements are calibrated to the degree of residual uncertainty. Residual uncertainty about a comparative effect is precisely the question that a comparative trial is designed to resolve. If uncertainty is deemed low in the absence of a comparative estimate, and that judgement is then used to justify not obtaining one, the reasoning becomes circular. It reflects an assessment of the adequacy of existing evidence rather than a general methodological principle.

The distinction between first-in-class devices and incremental modifications warrants the same scrutiny. Iterative modification is the mechanism through which entire classes of intracranial devices have entered clinical practice without a single randomised comparison against current best practice. Each modification is judged too small to justify a trial, and the cumulative consequence is technology deployed in tens of thousands of patients whose net effect on quality-adjusted survival has never been estimated. Learning curves and operator dependence do not exempt a device from this obligation; they can be accommodated by pragmatic multicentre randomised trials that reflect clinical reality, specify minimum operator experience and randomise at the level at which the treatment decision is made. This approach has already succeeded in neurointervention, as MR CLEAN demonstrates.^2^

The Delphi exercise the authors cite is a legitimate consensus mechanism, but consensus is not an epidemiological argument. A framework developed by clinicians with financial and advisory relationships with device manufacturers, and in several cases with the regulator, cannot substitute for a comparative estimate of effect.

For any device intended for patients who already have an established treatment, the minimum acceptable standard is a comparison against current best practice for the specific indication, ideally randomised. Below that threshold the counterfactual required to substantiate claims of safety or efficacy is absent, and no amount of adjudication, follow-up, transparency or regulatory endorsement can replace it. This is particularly relevant for wide-neck intracranial aneurysms, where microsurgical clipping, intrasaccular devices and flow-diverting stents are all available, yet their relative safety, effectiveness and durability remain uncertain. Randomised comparative studies are essential not only to establish whether a device is safe and effective, but also to determine which modality benefits specific patients and indications most. The ISAT trial provides a clear precedent, answering the relevant question for aneurysms amenable to both clipping and coiling and thereby informing practice.^3^ Newer devices deserve the same evaluation, so that they can be integrated into evidence-based guidelines.

## Data Availability

The manuscript references published data only, which is available online.
